# Inventory of arthropods (Arthropoda) through standardised protocols in the Teide National Park (Canary Islands, Spain)

**DOI:** 10.3897/BDJ.14.e181867

**Published:** 2026-06-02

**Authors:** Daniel Suárez, Manuel Cárdenas, Daniel Febles, Eduardo Jiménez-García, David Lugo, Carlos Ruiz, Nuria Macías-Hernández

**Affiliations:** 1 Departamento de Biología Animal, Edafología y Geología, Facultad de Ciencias, Universidad de La Laguna, La Laguna, Spain Departamento de Biología Animal, Edafología y Geología, Facultad de Ciencias, Universidad de La Laguna La Laguna Spain; 2 Island Ecology and Evolution Research Group, Instituto de Productos Naturales y Agrobiología, Consejo de Investigaciones Científicas, La Laguna, Spain Island Ecology and Evolution Research Group, Instituto de Productos Naturales y Agrobiología, Consejo de Investigaciones Científicas La Laguna Spain

**Keywords:** Arthropoda, Macaronesia, high-mountain ecosytem, new records, biodiversity

## Abstract

**Background:**

The Teide National Park is a high-mountain ecosystem characterised by a high temperature and solar radiation, together with low humidity. Aside from these extreme conditions, a rich biota inhabits there. In 1996, the first comprehensive inventory of arthropods was carried out and, since then, no further catalogue has been accomplished. The present work aims to update the current check-list of arthropods, as well as establishing sampling sites for further biomonitoring with standardised protocols.

**New information:**

A total of 615 taxa has been identified from the specimens recorded during the 2024–2025 field surveys, with 80 of them being new to the Teide National Park. This contribution highlights the outstanding biodiversity in an ecosystem characterised by extreme-conditions.

## Introduction

The Canary Islands are a volcanic archipelago located 96 km off the north-west coast of North Africa, comprising eight major islands. Tenerife is the largest (2,034 km^2^) and highest (3,718 m a.s.l.) island in the archipelago (Troll and Carracedo 2016). Together with La Palma, it is the only island of the archipelago where a high-mountain ecosystem develops. The summit of Tenerife is protected under the figure of 'National Park' and includes the Teide stratovolcano and a partially filled caldera (Caldera de Las Cañadas). Climate is characterised by a high annual and daily temperature variation, radiation is very intense, humidity is very low and dominant winds are dry (Martín-Esquivel et al. 2020). Due to these harsh environmental characteristics, its unique biota has developed morphophysiological adaptations to dryness. The legume summit scrub is the main potential vegetation on the summit of Tenerife (del Arco Aguilar and Rodríguez Delgado 2018), but more than 200 plant species have been reported (Martín-Esquivel et al. 2021). Invertebrate fauna is also rich, with 1,296 species of arthropods reported so far, 538 of them (41.5%) being endemics to the Canary Islands (Gobierno de Canarias 2025).

Although the summit of Tenerife has long attracted entomologists (e.g. Wollaston (1865), von Heyden (1872), Uyttenboogaart (1937), Wunderlich (1992)), the first and unique arthropod inventory to date was conducted between 1995 and 1996 (Oromí et al. 2002). In this first study, an extensive sampling in the Teide National Park was coducted, across two seasons (spring and autumn), applying a variety of sampling methods. A standardised protocol was applied to 21 sampling sites. In order to complete the faunal catalogue of the Teide National Park, additionally, 25 sites were sampled, but by conducting only active searching. Those sites were mainly associated with endemic and restricted plants, in order to look for specialist arthropods. Sampling sites were set mainly across the Caldera de Las Cañadas, but with some sampling sites on the Teide cone. As a result, 985 arthropod species were recorded (Oromí et al. 2002). Since this contribution, several studies increased the number of species reported (e.g. Dupont et al. (2003), Machado (2007), Macías-Hernández et al. (2016), Stüben and Astrin (2010), Suárez et al. (2025c)). However, 30 years after, no further inventory has been carried out.

## General description

### Purpose

The goal of this study is to characterise the arthropods fauna that inhabit the Teide National Park, updating the existing check-list and establishing a standardised protocol for future biomonitoring. Two standardised protocols were carried out. First, five sites were sampled following the COBRA protocol (Cardoso 2009). The second approach (passive sampling) encompassed the sampling in twelve sites across Las Cañadas caldera.

## Project description

### Title

Inventory of arthropods (Arthropoda) through standardised protocols in the Teide National Park (Canary Islands, Spain)

### Personnel

The project was conceived by Carlos Ruiz and Nuria Macías-Hernández.

Fieldwork (site selection and experimental setting): Daniel Suárez, Carlos Ruiz, Nuria Macías-Hernández.

Fieldwork (sample collection): Daniel Suárez, David Lugo, Eduardo Jiménez-García, Daniel Febles, Manuel Cárdena.

Taxonomists: Daniel Suárez, David Lugo, Eduardo Jiménez-García, Daniel Febles, Manuel Cárdena, David Brice.

Database management: Daniel Suárez.

Darwin Core databases: Daniel Suárez.

### Study area description

**Figure 1.**
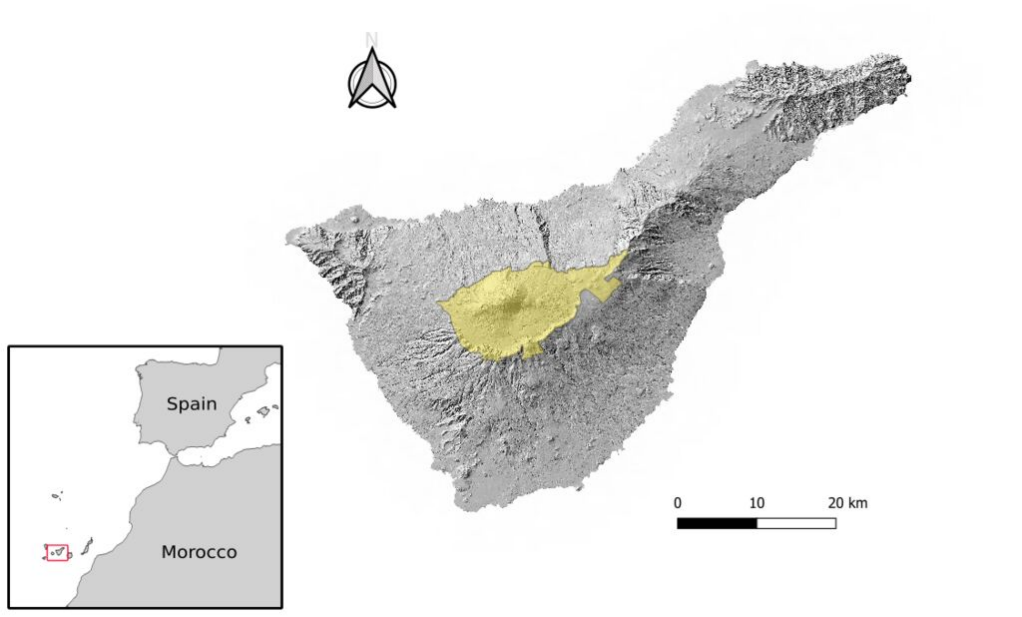
Map of Tenerife, with the surface area of the Teide National Park highlighted in yellow. Inset (left, bottom): location of the Canary Islands within the Atlantic Ocean (Tenerife highlighted in red).

The Teide National Park is located on the summit of the island of Tenerife (Canary Islands, Spain) covering ca. 189 km^2^. It encompasses the Teide cone and the Caldera de Las Cañadas (Fig. 1). The main vegetation type is a legume summit scrub constiting in the dominant legumes *Cytisus supranubius* and *Adenocarpus viscosus*, together with *Descurainia bourgaeana* and *Pterocephalus lasiospermus*.

## Sampling methods

### Sampling description

**Figure 2.**
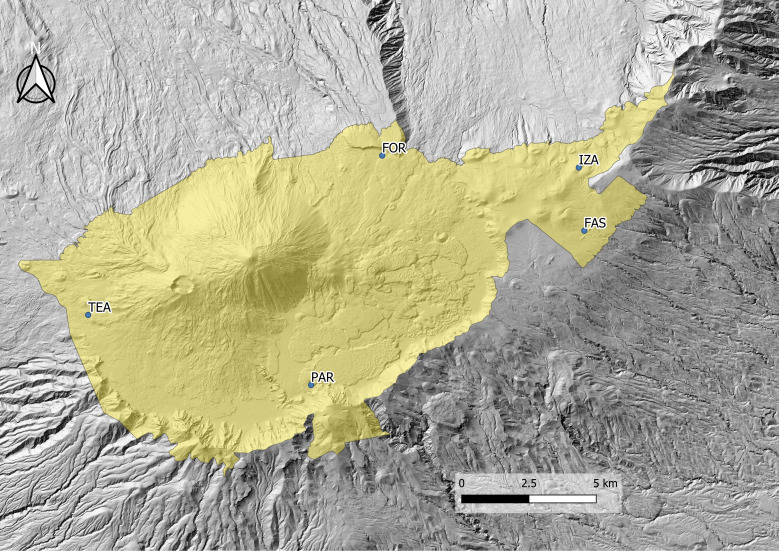
Map of the five sampling sites (dark blue dots) where the COBRA protocol was applied. The surface area of the Teide National Park is highlighted in yellow. Abbreviations: TEA: Cruz de Tea; PAR: Parador; FOR: Fortaleza; IZA: Izaña; FAS: Fasnia.

**Figure 3.**
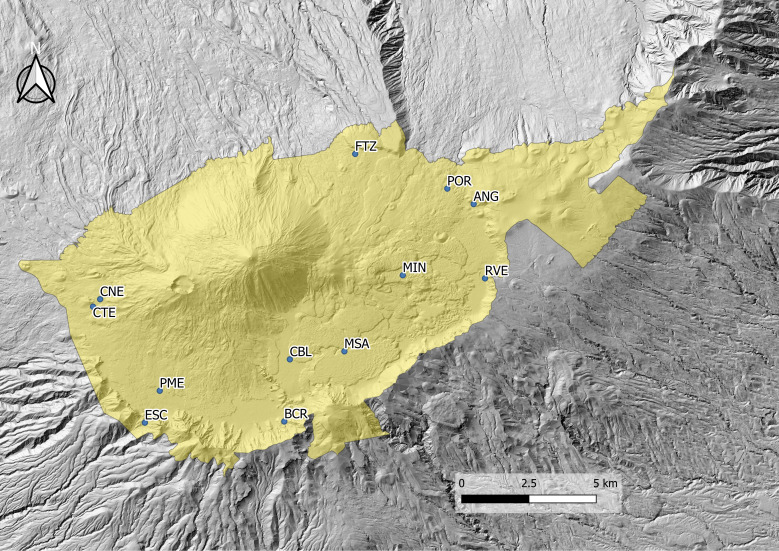
Map of the twelve sampling sites (dark blue dots) where passive sampling was applied. The surface area of the Teide National Park is highlighted in yellow. Abbreviations: CTE: Cruz de Tea; CNE: Cuevas Negras; ESC: Escobonal de Boca Tauce; PME: Cañada de Pedro Méndez; BCR: Barranco del Riachuelo; CBL: Cañada Blanca; MSA: Malpaís del Sanatorio; FTZ: Fortaleza; MIN: Minas de San José; POR: El Portillo; ANG: Arenas Negras; RVE: Risco Verde.

First, five sites were sampled following the COBRA protocol (Cardoso 2009) (Fig. 2). Sites were selected because they were near weather stations. In addition, one site was assigned to each of the four cardinal limits of the National Park. In the east, two sampling sites were included because this particular zone, located outside the Cañadas caldera, was not incorporated into the National Park until 1999. Therefore, the probability of expanding the faunal catalogue by including this sector was considered to be higher. The second approach (hereafter, "passive sampling") encompassed the sampling in twelve sites across Las Cañadas (Fig. 3), following the protocol of Oromí et al. (2002). High-elevation summits, such as the Teide cone, Montaña Blanca or Montaña Guajara, were excluded in order to prioritise sites with direct road access, thereby facilitating repeated sampling and ensuring consistency in field effort. The two sampling strategies partially overlap in methods, but not in sampling philosophy or effort. The COBRA protocol is strongly active and time-standardised, whereas the second strategy is predominantly passive, incorporating only a reduced active component to maintain comparability with previous studies and to complement passive traps. All the specimens collected were stored in vials with 99% ethanol.

**1. COBRA protocol**: the COBRA (Conservation Oriented Biodiversity Rapid Assessment) protocol was originally designed to monitor spiders in forested areas of Portugal (Cardoso 2009). It was later incorporated into the Global Island Monitoring Scheme (GIMS) as a standardised protocol for inventorying and monitoring arthropods in island ecosystems (Borges et al. 2018). Since its original formulation, although it has been adapted to sampling a variety of habitats (e.g. rainforests, tropical forests, dry shrublands, coastal wetlands) and arthropods (e.g. Diplopoda, Chilopoda, Coleoptera, Araneae) (Emerson et al. 2016, Malumbres‐Olarte et al. 2016, Borges et al. 2017, Borges et al. 2018, Salces-Castellano et al. 2021, Suárez et al. 2024). In order to adapt this protocol to an ecosystem with a high abundance of pollinators during the flowering peak, we have included two additional methods biased towards pollinators (i.e. pan traps and pollinator sampling). The following methods were included:

Vegetation beating: This technique involves striking branches and trunks above waist height to dislodge arthropods concealed within the foliage or resting on the branches. This is carried out using a wooden stick approximately 1 m long. A beating tray is placed beneath the vegetation to collect the falling specimens; it consists of a 1 × 1 m white cloth supported by two carbon-fibre rods crossing perpendicularly between opposite corners. The white surface facilitates the rapid detection and collection of arthropods. This technique was applied mainly to sample in the brooms *Cytisus supranubius* and *Adenocarpus viscosus* (Fabaceae). The sampling protocol included two hours during the day and two hours during the night.

Vegetation sweeping: Sweep-netting targets vegetation below waist height. It is performed with a sweeping net of 35 cm diameter and 68 cm depth. The net is vigorously swept across herbaceous vegetation and small shrubs to dislodge arthropods into the mesh. After sampling, the net contents are emptied on to a beating tray. This technique was applied mainly to sample in *Descurainia bourgaeana* (Brassicaceae) and *Pterocephalus lasiospermus* (Caprifoliaceae). The sampling protocol included two hours during the day and two hours during the night.

Active aerial searching: Involves the active search for arthropods within the sampling site, searching on shrub bark, beneath stones or moving across the ground. This method allows sampling of taxonomic or functional groups that are active during the night. The sampling protocol included four hours during the night.

Pollinator sampling: A butterfly net is used, consisting of a 46 cm metal ring, a 50 cm deep white nylon mesh and an extendable anodised aluminium handle (53–107 cm) coated with plastic. The method consists of approaching flowering plants and capturing individuals hovering over the blossoms or resting on flowers. The sampling protocol included two hours during the day.

Pan-traps: Pan traps are a passive capture method specifically designed for pollinating arthropods. The method consists of placing coloured trays (blue, white and yellow), which visually attract insects. Each tray is filled to half its depth with a solution of water and coconut betaine, prepared by adding one or two drops of coconut betaine per litre of water. Coconut betaine reduces the surface tension of the water, causing insects that land on the liquid to sink to the bottom of the trap without the possibility of escape. In addition, coconut betaine is odourless, thereby avoiding unintended attraction or repulsion effects. A total of nine trays (three per colour) were placed at each field site for eight hours.

Pitfall traps: Pitfall traps are a passive capture method consisting of plastic cups buried in the ground. Each cup measures 15 cm in height and 11 cm in diameter. The cups are placed in holes so that their rims are level with the soil surface, thus creating a continuous ground-level surface. They are filled to about one-quarter of their volume with propylene glycol, used as a preservative. Once the trap is set, two or more stones are placed beside the cup, supporting a larger stone that completely covers the trap, thereby protecting it from weather conditions. A total of 24 traps were placed at each field site for two weeks.

**2. Passive sampling**: Oromí et al. (2002) conducted different types of sampling, including active searching, pitfall, window and Malaise traps. In the current approximation, all the methods were replicated, except for window traps. Additionally, pan-traps and pollinator sampling were also carried out.

Limited active searching and targeted pollinator sampling were conducted as complementary methods. Although these latter techniques are not passive in a strict methodological sense, they represented a minor proportion of the total sampling effort and were included to supplement the predominantly passive sampling strategy, particularly to improve the detection of taxa poorly captured by traps.

Active aerial searching: This technique combines vegetation beating and sweeping (similar to the COBRA protocol). The sampling protocol included two hours during the day.

Pollinator sampling: The method consists of approaching flowering plants and capturing individuals hovering over the blossoms or lying on flowers (similar to the COBRA protocol). The sampling protocol included half an hour during the day.

Pan-traps: Pan traps are a passive capture method specifically designed for pollinating arthropods. Each tray is filled to half its depth with propylene glycol in order to avoid evaporation. A total of nine trays (three blue, three white and three yellow) were laid in the field for four days.

Pitfall traps: Pitfall traps are a passive capture method consisting of plastic cups buried in the ground. In this approach the traps are filled with Turquin liquid (1 litre of beer + 5 ml of acetic acid). The combination of beer and acetic acid acts as an attractant for fauna. For sample preservation, instead of using chloral hydrate, ca. 20 ml of propylene glycol was added. In addition, a vial with chicken liver acting as a bait is placed inside the trap as an additional lure. This method allows the collection of a large number of individuals in a short period of time. A total of six traps per site were laid in the field for four days.

Malaise traps: The Malaise trap (Townes style) is an interception method for flying arthropods. It consists of a system of mesh panels and poles arranged in the shape of a tent. Arthropods intercepted by the mesh, due to negative geotropism, ascend inside the trap until reaching the only available opening, which leads into a collecting jar mounted on a vertical wooden pole. The jar is filled to one-quarter of its volume with propylene glycol. One trap per site was laid in the field for four days.

## Geographic coverage

### Description

Teide National Park (Tenerife, Canary Islands, Spain).

### Coordinates

28.18713192 and 28.33949168 Latitude; -16.48169166 and -16.732517 Longitude.

## Taxonomic coverage

### Description

Arthropoda.

## Temporal coverage

**Data range:** 2024-5-01 – 2025-6-30.

### Notes

Sampling was carried out during two years. In 2024, there were two seasonal samplings, during spring (May and June) and autumn (September and October). In 2025, the sampling was done during spring (May and June).

## Collection data

### Collection name

Invertebrate collection of the Department of Zoology of the University of La Laguna.

### Collection identifier

DZUL.

### Specimen preservation method

All specimens are preserved in 99% ethanol and stored in a freezer (-20ºC).

## Usage licence

### Usage licence

Other

### IP rights notes

Creative Commons Attribution 4.0 International (CC-BY-4.0).

## Data resources

### Data package title

Arthropods from the National Park of El Teide.

### Resource link

https://doi.org/10.15470/zvhfl6

### Number of data sets

2

### Data set 1.

#### Data set name

Teide Arthropod Occurrences (Passive sampling).

#### Data format

Darwin Core.

#### Download URL

https://www.gbif.org/dataset/b8c4509c-c3a7-420b-ab03-b92401d3d19c

#### Description

Counts of arthropods collected in the Canary Islands (Tenerife) using passive traps (pan, pitfall, Malaise, net and interception) during surveys carried out in 2024 and 2025 at 12 localities across the Teide National Park (Suárez et al. 2025a). Each row represents a taxon counted in a single trap sample. Variables include locality code, coordinates, sampling date and method, taxonomic identification to genus or species and individual count.

**Data set 1.** 

Column label	Column description
id	Identifier of the sampling event.
type	Nature of the record.
modified	Year of the last modification.
language	Language of the dataset.
licence	Licence of the dataset.
datasetID	Identifier of the dataset including DOI.
institutionCode	The name (or acronym) in use by the institution having custody of the object(s) or information referred to in the record.
collectionCode	The name of the collection from which the record was derived.
datasetName	The name of the dataset from which the record was derived.
basisOfRecord	The specific nature of the data record (in this case “PreservedSpecimen”), a subtype of dcterms:type; recommended to use the Darwin Core Type Vocabulary. dwc.tdwg.orgdocs.google.com.
occurrenceID	An identifier for the occurrence.
catalogNumber	Code given by the institution.
individualCount	Number of specimens in the occurrence.
occurrenceStatus	Status of the ocurrence.
eventDate	The date during which the event occurred.
year	The year during which the event occurred.
month	The month during which the event occurred.
day	The day during which the event occurred.
samplingProtocol	The name of, reference to, or description of the method or protocol used during a sampling event.
locationID	An identifier for the location.
continent	The name of the continent in which the location occurs.
islandGroup	The name of the island group in which the location occurs.
island	The name of the island in which the location occurs.
country	The name of the country in which the location occurs.
countryCode	The standard code for the country in which the location occurs.
stateProvince	The name of the province in which the location occurs.
locality	Specific description of the location of the study sites.
decimalLatitude	The geographic latitude (in decimal degrees) of the geographic centre of the sampling plot.
decimalLongitude	The geographic longitude (in decimal degrees) of the geographic centre of the sampling plot.
geodeticDatum	The geodetic datum upon which the geographic coordinates given in decimal latitude and decimal longitude are based.
coordinateUncertaintyInMetres	The horizontal distance (in metres) from the given decimal latitude and decimal longitude describing the smallest circle containing the whole of the location.
identifiedBy	Name or names of the observer/s identifying the taxon.
scientificName	Scientific name of the species.
kingdom	Kingdom of the species.
order	Taxonomic order of the species.
family	Taxonomic family of the species.
genus	Taxonomic genus of the species.
specificEpithet	Taxonomic epithet of the species.
taxonRank	The taxonomic rank of the most specific name in the scientificName (e.g. “species”, “subspecies”).
verbatimIdentification	A string representing the taxonomic identification as it appeared in the original record.
identificationQualifier	A brief phrase or a standard term ("cf.", "aff.") to express the determiner's doubts about the identification.
eventID	An identifier for the set of information associated with a event (something that occurs at a place and time).
eventTime	The time or interval during which an event occurred.
samplingEffort	The amount of effort expended during an event.
habitat	A category or description of the habitat in which the event occurred.
minimumElevationInMetres	The lower limit of the range of elevation (altitude, usually above sea level), in metres.
maximumElevationInMetres	The upper limit of the range of elevation (altitude, usually above sea level), in metres.
recordedBy	A person, group or organisation responsible for recording the original occurrence.

### Data set 2.

#### Data set name

Teide Arthropod Occurrences (COBRA protocol).

#### Data format

Darwin Core.

#### Download URL

https://www.gbif.org/dataset/200df665-a0f4-4802-87f8-b238dd8c0591

#### Description

Flat table of terrestrial arthropod occurrences collected in 2024–2025 from five sites on Tenerife, Canary Islands, using standard COBRA sampling protocols (Suárez et al. 2025b). Each row holds the unique field code, site information, coordinates, collection date, sampling method, taxon identification, number of individuals and name of the identifier.

**Data set 2.** 

Column label	Column description
id	Identifier of the sampling event.
type	Nature of the record.
modified	Year of the last modification.
language	Language of the dataset.
licence	Licence of the dataset.
datasetID	Identifier of the dataset including DOI.
institutionCode	The name (or acronym) in use by the institution having custody of the object(s) or information referred to in the record.
collectionCode	The name of the collection from which the record was derived.
datasetName	The name of the dataset from which the record was derived.
basisOfRecord	The specific nature of the data record (in this case “PreservedSpecimen”), a subtype of dcterms:type; recommended to use the Darwin Core Type Vocabulary. dwc.tdwg.orgdocs.google.com.
occurrenceID	An identifier for the occurrence.
recordNumber	Code given by the institution.
individualCount	Number of specimens in the occurrence.
occurrenceStatus	Status of the ocurrence.
eventDate	The date during which the event occurred.
year	The year during which the event occurred.
month	The month during which the event occurred.
day	The day during which the event occurred.
samplingProtocol	The name of, reference to, or description of the method or protocol used during a sampling event.
locationID	An identifier for the location.
islandGroup	The name of the island group in which the location occurs.
island	The name of the island in which the location occurs.
country	The name of the country in which the location occurs.
countryCode	The standard code for the country in which the location occurs.
locality	Specific description of the location of the study sites.
decimalLatitude	The geographic latitude (in decimal degrees) of the geographic centre of the sampling plot.
decimalLongitude	The geographic longitude (in decimal degrees) of the geographic centre of the sampling plot.
geodeticDatum	The geodetic datum upon which the geographic coordinates given in decimal latitude and decimal longitude are based.
coordinateUncertaintyInMetres	The horizontal distance (in metres) from the given decimal latitude and decimal longitude describing the smallest circle containing the whole of the location.
identificationQualifier	Additional information to the identification.
identifiedBy	Name or names of the observer/s identifying the taxon.
scientificName	Scientific name of the species.
kingdom	Kingdom of the species.
order	Taxonomic order of the species.
family	Taxonomic family of the species.
subfamily	Taxonomic subfamily of the species.
genus	Taxonomic genus of the species.
specificEpithet	Taxonomic epithet of the species.
taxonRank	The taxonomic rank of the most specific name in the scientificName (e.g. “species”, “subspecies”).
verbatimIdentification	A string representing the taxonomic identification as it appeared in the original record.
eventID	An identifier for the set of information associated with an event (something that occurs at a place and time). May be a global unique identifier or an identifier specific to the dataset.
eventTime	The time or interval during which an event occurred.
samplingEffort	The amount of effort expended during an event.
habitat	A category or description of the habitat in which the event occurred.
minimumElevationInMetres	The lower limit of the range of elevation (altitude, usually above sea level), in metres.
maximumElevationInMetres	The upper limit of the range of elevation (altitude, usually above sea level), in metres.
recordedBy	A person, group or organisation responsible for recording the original occurrence.

## Additional information

**Figure 4.**
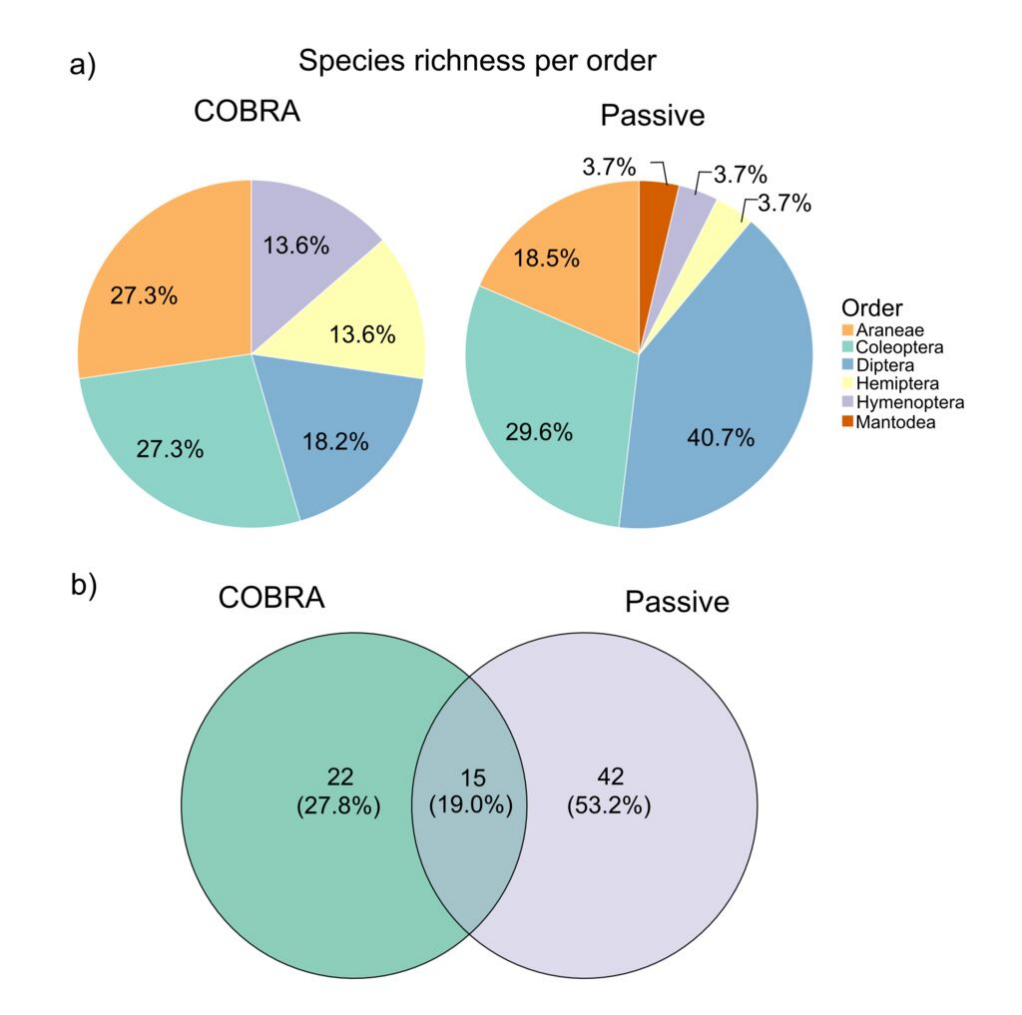
Comparison of the two sampling approaches regarding new records. **a**) Pie chart per sampling approach of the species richness at the order scale; **b**) Venn diagram of the number of exclusive and shared species between sampling approaches.

In the first approximation (COBRA protocol), a total of 23,998 individuals belonging to 403 taxa were collected. With the second approximation (passive sampling), a total of 21,592 individuals belonging to 474 taxa were sampled. Pooling both approaches, a total of 45,590 specimens belonging to 615 taxa were identified. Amongst these taxa, 79 of them are new records for the Teide National Park, with eight of them being also new records for the island of Tenerife and 14 are new to the Canary Islands (Table 1). Amongst these new records, in the COBRA protocol, the orders that accounted for a higher number of species were Araneae and Coleoptera (27.3% each), followed by Diptera (18.2%) and Hemiptera and Hymenoptera (13.6% each). However, in the passive sampling, the order with the highest number of new records was Diptera (40.7%), followed by Coleoptera (29.6%), Araneae (18.5%) and Hemiptera, Hymenoptera and Mantodea (3.7% each) (Fig. 4a). Regarding the number of new records by sampling approach, 42 species (53.2%) were exclusive to the passive sampling and 22 (27.8%) were exclusive to the COBRA protocol. In contrast, 15 (19.0%) new records were found in both approaches (Fig. 4b).

Most of the new faunistic records obtained in this study correspond to Diptera, a group that has been historically under-represented in high-elevation surveys of Teide National Park and that have been now identified by a specialist taxonomist (David Brice). Additional new records include other orders, such as spiders or beetles, several of which appearing to be extending their upper altitudinal limits, i.e. are typically found in low-altitude xeric areas or mid-altitude humid forests, based on chorological information available on the Biodiversity Databank of the Canary Islands (https://www.biodiversidadcanarias.es/biota/). However, their current relative abundances remain low, suggesting early stages of range expansion rather than established populations. Regarding sampling strategies, both protocols proved to be complementary: the COBRA protocol is better suited for standardised, long-term monitoring and robust temporal or spatial comparisons, particularly for arthropods associated with vegetation, whereas the predominantly passive sampling provides a cost-effective overview of community composition with reduced field effort and is especially efficient at capturing flying taxa, such as Diptera and Hymenoptera, making it advantageous for rapid inventories and baseline assessments in the Park. Therefore, the differences observed between sampling methods are best interpreted as complementary detectability biases rather than methodological inconsistencies. Using both approaches together enhances overall community coverage and reduces the likelihood that absences are driven solely by method-specific detection limitations.

## Figures and Tables

**Table 1.** New records for the Teide National Park.

Order	Family	Species	New record
Araneae	Araneidae	*Agalenatea redii* (Scopoli, 1763)	Teide
Araneae	Araneidae	*Araneus bufo* (Denis, 1941)	Teide
Araneae	Araneidae	*Araniella maderiana* (Kulczynski, 1905)	Teide
Araneae	Araneidae	*Hypsosinga albovittata* (Westring, 1851)	Teide
Araneae	Araneidae	*Mangora acalypha* (Walckenaer, 1802)	Teide
Araneae	Gnaphosidae	*Macarophaeus insignis* Wunderlich, 2011	Teide
Araneae	Linyphiidae	*Tenuiphantes canariensis* (Wunderlich, 1987)	Teide
Araneae	Salticidae	*Ballus chalybeius* (Walckenaer, 1802)	Teide
Araneae	Salticidae	*Chalcoscirtus infimus* (Simon, 1868)	Teide
Araneae	Salticidae	*Pellenes arciger* (Walckenaer, 1837)	Tenerife
Coleoptera	Coccinellidae	*Myrrha octodecimguttata* (Linnaeus, 1758)	Teide
Coleoptera	Coccinellidae	*Nephus nigricans* Weise, 1879	Tenerife
Coleoptera	Curculionidae	*Ceutorhynchus jucundus* Colonnelli, 2005	Tenerife
Coleoptera	Curculionidae	*Lixus machadoi* Krátký, Stüben & Turner, 2023	Teide
Coleoptera	Latridiidae	*Corticaria fagi* Wollaston, 1854	Tenerife
Coleoptera	Nitidulidae	*Afrogethes canariensis* (Kirejtshuk, 1997)	Teide
Coleoptera	Nitidulidae	*Carpophilus bifenestratus* Murphy, 1864	Teide
Coleoptera	Nitidulidae	*Carpophilus zeaphilus* Dobson, 1969	Canary Islands
Coleoptera	Nitidulidae	*Xenostrongylus canariensis* Wollaston, 1854	Teide
Coleoptera	Phalacridae	*Phalacrus coruscus* (Panzer, 1797)	Teide
Coleoptera	Staphylinidae	*Aleochara funebris* Wollaston, 1864	Teide
Coleoptera	Staphylinidae	*Atheta fungi* (Gravenhorst, 1806)	Teide
Coleoptera	Staphylinidae	*Tachyporus nitidulus* (Fabricius, 1781)	Teide
Diptera	Agromyzidae	*Liriomyza flaveola* (Fallén, 1823)	Teide
Diptera	Anthomyiidae	*Napomyza lateralis* (Fallén, 1823)	Canary Islands
Diptera	Bombyliidae	*Phthiria antiqua* Báez, 1985	Teide
Diptera	Bombyliidae	*Thyridanthrax indigenus* (Becker, 1908)	Teide
Diptera	Calliphoridae	*Calliphora vomitoria* (Linnaeus, 1758)	Teide
Diptera	Ceratopogonidae	*Dasyhelea albidipes* (Santos Abreu, 1918)	Teide
Diptera	Ceratopogonidae	*Forcipomyia pallidipes* Santos Abreu, 1918	Tenerife
Diptera	Ceratopogonidae	*Forcipomyia sahariensis* Kieffer, 1923	Canary Islands
Diptera	Chloropidae	*Oscinimorpha tenuirostris* (Duda, 1933)	Canary Islands
Diptera	Chloropidae	*Tricimba humeralis* (Loew, 1858)	Teide
Diptera	Drosophilidae	*Drosophila latifrons* Adams, 1905	Canary Islands
Diptera	Drosophilidae	*Drosophila obscura* Fallén, 1823	Canary Islands
Diptera	Drosophilidae	*Drosophila suzukii* (Matsumura, 1931)	Canary Islands
Diptera	Drosophilidae	*Lordiphosa andalusiaca* (Strobl, 1906)	Tenerife
Diptera	Drosophilidae	*Scaptomyza pallida* Zetterstedt, 1847	Teide
Diptera	Heleomyzidae	*Eccoptomera microps* (Meigen, 1830)	Canary Islands
Diptera	Heleomyzidae	*Heleomyza modesta* Meigen, 1835	Canary Islands
Diptera	Heleomyzidae	*Pseudoleria pectinata* (Loew, 1872)	Canary Islands
Diptera	Hybotidae	*Platypalpus strakai* Chvála, 1989	Canary Islands
Diptera	Limoniidae	*Atypophthalmus quinquevittatus* (Santos Abreu, 1923)	Teide
Diptera	Limoniidae	*Dicranomyia intermedia* Santos Abreu, 1923	Teide
Diptera	Milichiidae	*Leptometopa rufifrons* Becker, 1903	Teide
Diptera	Milichiidae	*Milichia mixta* Becker, 1907	Teide
Diptera	Muscidae	*Muscina levida* (Harris, [1780])	Teide
Diptera	Muscidae	*Synthesiomyia nudiseta* (Van der Wulp, 1833)	Teide
Diptera	Mycetophilidae	*Docosia gilvipes* (Walker, 1856)	Tenerife
Diptera	Odiniidae	*Odinia trifida* Carles-Tolrá, 1996	Canary Islands
Diptera	Phoridae	*Phora atra* (Meigen, 1804)	Teide
Diptera	Psychodidae	*Mormia tenebrosa* (Satchell, 1955)	Teide
Diptera	Psychodidae	*Sergentomyia fallax* (Parrot, 1921)	Teide
Diptera	Sarcophagidae	*Blaesoxipha lapidosa* (Pape, 1996)	Tenerife
Diptera	Sarcophagidae	*Miltogramma aurifrons* Dufour, 1850	Teide
Diptera	Sarcophagidae	*Sarcophaga crassipalpis* Macquart, 1839	Teide
Diptera	Sarcophagidae	*Sarcophaga tibialis* Macquart, 1850	Teide
Diptera	Sarcophagidae	*Sarcophila latifrons* (Fallén, 1817)	Teide
Diptera	Sarcophagidae	*Taxigramma heteroneura* (Meigen, 1830)	Teide
Diptera	Scatopsidae	*Coboldia fuscipes* (Meigen, 1830)	Teide
Diptera	Scatopsidae	*Scatopse notata* (Linnaeus, 1758)	Teide
Diptera	Scatopsidae	*Swammerdamella brevicornis* (Meigen, 1830)	Canary Islands
Diptera	Syrphidae	*Episyrphus balteatus* (De Geer, 1776)	Teide
Diptera	Syrphidae	*Eupeodes corollae* (Fabricius, 1794)	Teide
Diptera	Syrphidae	*Melanostoma incompletum* Becker, 1908	Teide
Diptera	Syrphidae	*Scaeva selenitica* (Meigen, 1822)	Canary Islands
Diptera	Tachinidae	*Ceracia mucronifera* Rondani, 1865	Teide
Diptera	Tachinidae	*Chetogena acuminata* Rondani, 1859	Teide
Diptera	Tipulidae	*Tipula multipincta* Becker, 1908	Teide
Diptera	Trixoscelidae	*Trixoscelis puncticornis* (Becker, 1907)	Teide
Hemiptera	Alydidae	*Camptopus lateralis* (Germar, 1817)	Teide
Hemiptera	Hebridae	*Merragata hebroides* Buchanan-White, 1877	Teide
Hemiptera	Miridae	*Perenotus stysi* (Ribes, Pagola-Carte & Heiss, 2008)	Teide
Hemiptera	Tingidae	*Acalypta parvula* (Fallén, 1807)	Teide
Hymenoptera	Braconidae	*Chelonus oculator* (Fabricius, 1775)	Teide
Hymenoptera	Braconidae	*Cotesia vanessae* (Reinhard, 1880)	Teide
Hymenoptera	Formicidae	*Cardiocondyla mauritanica* Forel, 1890	Teide
Hymenoptera	Megachilidae	*Dioxys atlanticus* Saunders, 1904	Teide
Mantodea	Empusidae	*Hypsicorypha gracilis* (Burmeister, 1838)	Teide
